# Glaucoma Classification Using a NFNet-Based Deep Learning Model with a Customized Hybrid Attention Mechanism

**DOI:** 10.3390/diagnostics16050815

**Published:** 2026-03-09

**Authors:** Sandeep Angara, Loc Tran, Jongwoo Kim

**Affiliations:** Lister Hill National Center for Biomedical Communications, National Library of Medicine, National Institutes of Health, Bethesda, MD 20892, USA; sandeepangara@gmail.com (S.A.); loctransps@gmail.com (L.T.)

**Keywords:** glaucoma, deep learning, normalization-free ResNet, hybrid attention, channel attention, spatial attention

## Abstract

**Background/Objectives:** Glaucoma is a leading cause of irreversible blindness worldwide, making accurate and efficient detection methods essential. One primary concern with glaucoma is that it often presents no early symptoms. Vision loss typically begins at the periphery and progresses unnoticed until it significantly affects central vision. Due to this gradual and usually silent progression, early detection through regular eye exams is vital for preventing permanent vision loss. **Methods:** In this study, we propose a hybrid attention mechanism that recalibrates feature maps from the feature extractor for glaucoma detection. We explored normalization-free ResNet (NF-ResNet) architectures to evaluate the proposed attention mechanism, specifically NF-ResNet-26, NF-ResNet-50, and NF-ResNet-101, in comparison to baseline state-of-the-art ResNet variants. Our approach was evaluated on three publicly available glaucoma datasets, LAG, EyePACS, and BrG, to differentiate between normal and glaucomatous from fundus images. **Results:** The experimental results demonstrate that our proposed hybrid attention module, combined with normalization-free architectures, significantly enhances performance compared to state-of-the-art ResNet variants. The proposed attention model based on the normalization-free ResNet-50 achieved an accuracy of 0.9394 on the LAG dataset, 0.9117 on the EyePACS dataset, and 0.9020 on the BrG dataset. When evaluated on the combined dataset, the model achieved an accuracy of 0.9193, sensitivity of 0.9182, and specificity of 0.9202. **Conclusions:** The results from these representative datasets for glaucoma detection highlight the exceptional performance of our attention module, establishing it as a highly competitive classification model in the field of glaucoma detection.

## 1. Introduction

Glaucoma is a condition that damages the eye’s optic nerve and can result in vision loss and even blindness. It damages the optic nerve that sends images to the brain. As the world’s population ages, glaucoma is becoming an increasing cause of blindness. According to the WHO [1], glaucoma is the second leading cause of blindness globally, after cataracts. However, glaucoma presents a more significant challenge since the blindness it causes is irreversible. It has been estimated that 60.5 million people were affected by glaucoma in 2010, with a projected number of 112 million by 2040 [2]. Many people with the disease are unaware of it, even in developed countries. This lack of awareness can lead to advanced disease and significant vision loss at the time of diagnosis. So, finding the disease early and acting is crucial for reducing vision loss due to glaucoma.

Glaucoma occurs when the optic nerve becomes damaged, leading to a gradual deterioration that results in blind spots in vision. This damage is often associated with increased intraocular pressure, caused by a buildup of aqueous humour that usually flows inside the eye. Under normal conditions, this fluid drains through the trabecular meshwork located at the junction of the iris and cornea. The cornea is vital for vision, allowing light to enter the eye. However, intraocular pressure can rise if the eye produces too much fluid or if the drainage system malfunctions. In individuals with glaucoma, elevated pressure or reduced blood flow to the optic nerve can cause nerve fibres to die. This leads to enlargement of the optic cup relative to the optic disc due to the loss of supporting tissue. While optic nerve cupping is present in healthy individuals and those with glaucoma, those with the condition tend to have a larger cup-to-disc ratio. A cup-to-disc ratio greater than 0.6 is generally considered suspicious for glaucoma.

Currently, glaucoma diagnosis and monitoring require a complete eye examination, additional testing, and gathering a slew of data, which can be challenging to interpret. Moreover, ophthalmologists use multiple manual methods to diagnose glaucoma, including gonioscopy, pachymetry, tonometry, and perimetry [3]. However, manual assessment methods are very time-consuming and largely depend on the availability of ophthalmologists. Furthermore, there is a significant overlap in the ocular features of normal subjects and patients with early glaucoma. For these reasons, there is interest in developing complementary techniques—such as artificial intelligence (AI) systems—to diagnose glaucoma effectively.

Artificial intelligence (AI) has revolutionized the detection and diagnosis of retinal diseases in recent years, mainly through advances in deep learning and computer vision. AI algorithms, such as convolutional neural networks (CNNs), have been trained on large datasets of retinal images to accurately identify conditions like glaucoma, diabetic retinopathy, age-related macular degeneration, and cataracts. These models can quickly analyze medical images like optical coherence tomography (OCT) scans and fundus photographs, detecting subtle abnormalities the human eye may overlook. Using AI-powered tools improves diagnostic accuracy and enables early detection, a crucial factor in preventing vision loss. Furthermore, AI systems have the potential to enhance access to eye care, particularly in remote or underserved areas, by providing real-time screening and decision support for healthcare professionals. This integration of AI into ophthalmology is setting a new standard for personalized and efficient patient care, potentially benefiting society at large.

Das et al. [4] proposed a novel cascaded attention module (CAM) consisting of a triplet channel attention block (TCAB) and a spatial attention block (SAB) on top of a backbone network for glaucoma classification. A cascaded attention module (CAM) mainly consists of two components, namely a triplet channel attention block (TCAB) and a spatial attention block (SAB), connected in a cascaded manner. The CAM builds feature relationships along channel, cross-channel, and spatial dimensions and facilitates extracting rich discriminative features from the lesion regions of fundus images. The study clearly shows an improvement in performance on Harvard Dataset V1 and LMG datasets by adding the CAM on top of the backbone network. Various backbone networks have been tested, and DenseNet-121 with CAM performed better than the other backbone networks; the model with DenseNet-121 achieved an accuracy of 85.34% on the Harvard dataset and 83.85% on the LMG dataset.

Latif et al. [5] proposed a two-phase optic disc localization and glaucoma diagnosis network (ODGNet). A shallow convolutional neural network was incorporated in the first phase to localize optic discs from fundus images. In the later phase, AlexNet, ResNet, and VGGNet were used to diagnose glaucoma. The models were trained using transfer learning with pre-trained weights on five retinal datasets: ORIGA, HRF, DRION-DB, DR_HAGIS, and RIM_ONE.

Fan et al. [6] used a vision transformer deep learning technique, data-efficient image transformer (DeiT), and ResNet-50 trained on fundus images from an ocular hypertension treatment study to detect primary open-angle glaucoma. This study comprehensively explored the generalizability and explainability of vision transformers to detect glaucoma using fundus photographs. The extensive experiments suggest that vision transformers (ViT) generalize well to the eyes of individuals of Chinese, Japanese, Spanish, African and European fundus photographs. Furthermore, ViT focused on localized features of the neuroretinal rim, often used in the clinical management of glaucoma.

Silva et al. [7] developed a tool for classifying diabetic retinopathy from retinal images. They utilized the pre-trained weights of a Normalizer-Free Neural Network (NFNet) as a feature extractor. Subsequently, they incorporated classifiers such as Naïve Bayes, k-Nearest Neighbours (kNN), Multi-Layer Perceptron (MLP), Ordered Probabilistic Forest (OPF), and Support Vector Machine (SVM) to classify the retinal images as either healthy or affected by diabetic retinopathy.

Yang et al. [8] proposed a Mobilenetv2 lightweight network for retinopathy classification, incorporating spatial and channel reconstruction convolution and convolutional block attention module mechanisms into the framework. Incorporating attention modules helped reduce the features by limiting the redundant features and enhanced the feature representation ability for classification improvement. This study established a retinal dataset from ophthalmic clinical images and classified them based on retinal B-Scan image features, which were obtained from the First Affiliated Hospital of Zhengzhou University. This dataset has five classes: AMD, DME, DRUSEN, MNV, and Normal.

Chakraborty et al. [9] presented an attention-aided DenseNet-121 for detecting glaucoma from fundus images. They introduced an amalgamation of two attention modules, the convolutional block attention module (CBAM) and the Channel recalibration module (CRM), on the extracted features from DenseNet-121. The CBMA helps highlight relevant spatial and channel features extracted by the feature extractor. The channel recalibration module further enriches the features by utilizing the edge information and the spatial dimension’s statistical features. RIM-ONE and ACRIMA datasets have been used in this study.

Das et al. [10] proposed AES-NET for effective multi-stage glaucoma classification using fundus images. The fundus images are passed into a backbone network, which can be any ImageNet pre-trained CNN. The feature maps from the last convolution layer of the backbone network are then passed to the spatial adapter module. The spatial adapter module allows the adjustment of its spatial representations per the target task’s requirements. After the spatial adapter module, the features are passed to an enhanced self-attention module to selectively extract more detailed-lesion features from fundus images. Harvard Dataverse V1 and LMG datasets have been used in the study.

Most of the studies discussed have used small-sized datasets with high-quality fundus images. Moreover, minimal studies have been done on normalization-free networks. In this study, we used three datasets of different qualities to train the models and for robust evaluation of the proposed attention modules along with normalization-free architectures.

In this paper, we:

- Propose a hybrid attention module that combines spatial and channel attention to calibrate features for glaucoma classification from fundus images.
- Explore normalization-free networks as feature extractors in combination with hybrid attention modules. Various hybrid attention modules were utilized alongside distinct versions of normalization-free ResNets (NF-ResNets) [11] models.
- Analyze our hybrid attention module with NFNets across three datasets: LAG [12], BrG [13], and EyePacs [14].
- Further evaluate the proposed attention modules using five-fold cross-validation on the combined LAG, BrG, and EyePACS datasets and conduct comparisons with state-of-the-art (SOTA) models.

## 2. Materials and Methods

### 2.1. Datasets

**Figure 1:**
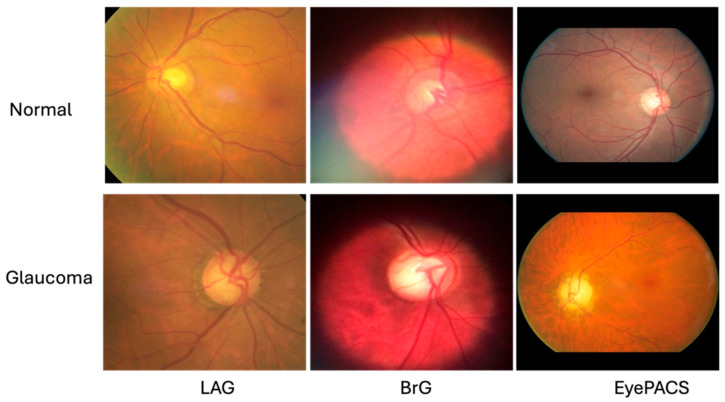
Sample images of normal and glaucoma from LAG, BrG, and EyePACS datasets.

We used three publicly datasets for training and evaluating the models robustly. Samples of images of standard and glaucomatous are displayed in Figure 1.

LAG [12]: The subset of the LAG database has been used for experiments and comprises 4844 fundus images, including 1711 samples confirmed to have glaucoma and 3143 normal samples sourced from Beijing Tongren Hospital. Each image has been annotated by certified glaucoma specialists, who consider various factors, including morphological and functional analyses such as intraocular pressure, visual field loss, and manual assessment of the optic disc to annotate the fundus image. The training set includes 3064 samples, the validation set contains 802 samples, and the test dataset consists of 991 samples.

EyePACS-light (v2) [13]: This enhanced glaucoma dataset comprises a balanced selection of standardized fundus images from the Rotterdam EyePACS AIROGS dataset. It includes data collected from 500 diverse sites with varying ethnicities. The dataset is organized into training, validation, and testing folders, containing 4000 (~84%), 385 (~8%), and 385 (~8%) fundus images across each class, respectively.

BrG [14]: The fundus images in this dataset were collected from Hospital de Olhos (HO) in Minas Gerais (MG) and Policlinica de Unai, MG, Brazil. The dataset includes 2000 images, with 1000 representing glaucoma cases and 1000 normal cases. A group of 1000 volunteers had their eye fundus images captured for both left and right eyes. The images were captured using a smartphone attached to a panoptic ophthalmoscope, utilizing the iExaminer app installed on the smartphone.

In our initial experiments, the LAG and EyePACS-light datasets were originally divided into training, validation, and test sets, whereas the BrG dataset did not include predefined splits. For the BrG dataset, we performed a patient-level split, allocating 70% of the data for training and 30% for testing. From the training set, 20% was further used as a validation set.

To conduct a more robust model evaluation, we adopted a five-fold cross-validation strategy on the combined dataset. Each individual dataset was first divided into five folds. For each cross-validation iteration, four folds from each dataset were merged to form the training set, while the remaining folds were reserved for testing. The merged training data were then randomly split into 80% for training and 20% for validation, while preserving class balance. This approach enables a thorough assessment across multiple datasets while maintaining balanced class distributions in both the training and validation sets. The model was trained and evaluated separately in each of the five cross-validation folds.

### 2.2. Deep Learning Architecture

**Figure 2:**
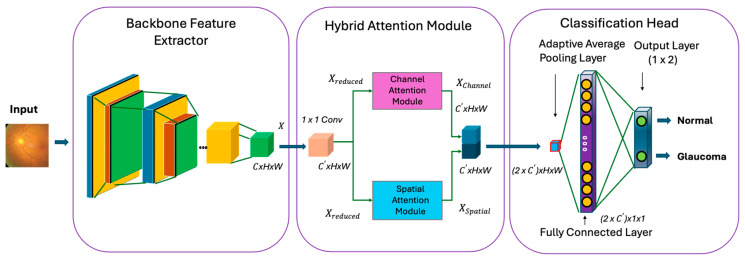
The overall architecture of the proposed framework for automated classification of glaucoma.

The proposed deep learning architecture comprises a backbone network, a hybrid attention module, and a classification head, as shown in Figure 2. The backbone network captures hierarchical features from raw input images. These extracted features provide a strong foundation for downstream tasks, allowing complex information to be represented in a compact and useful format. The hybrid attention module is designed to selectively enhance important information from the feature maps that the feature extractor provides. Subsequently, the recalibrated features are passed through a classification head, which includes a global average pooling layer and a fully connected layer to perform glaucoma classification.

#### 2.2.1. Backbone

As illustrated in the first block in Figure 2, we provide the fundus image to pass as an input through the backbone network. The features are extracted from the last convolutional layer of the backbone network. This study considered ResNet [15] and NF-ResNet variants as backbone networks.

The choice of the ResNet architecture was driven by both its theoretical foundation and practical experimental considerations. NFNet’s normalization-free design was originally an extension of the residual learning approach, making ResNet a logical and structurally consistent backbone for this study. Using ResNet enables a fair comparison among standard residual models, normalization-free variants, and our hybrid attention mechanism, while reducing architectural confounds. This selection was primarily dictated by methodological reasoning rather than solely by ablation outcomes, with subsequent experiments confirming the effectiveness of our proposed modifications.

In these experiments, we explored networks with 26, 50, and 101 layers for extreme analysis of the models.

#### 2.2.2. Hybrid Attention Module

We propose an attention module that helps to recalibrate the features extracted from the feature extractor to help capture rich semantic information from fundus images. The hybrid attention module comprises spatial and channel attention as shown in the second block in Figure 2. The hybrid attention module is placed between the feature extractor and the classification, designed to selectively enhance the critical information from feature maps at both channel and spatial levels.

Let X ∈ the ${R\;}^{C\;\times \;H\;\times \;W}$ represent the feature map from the feature extractor, where *C* represents channels, height *H*, and width *W*. Before passing the features to the attention modules, a pointwise convolution is applied to reduce the number of channels and is controlled by the reduction ratio (r). The reduction ratio controls the degree of channel compression while retaining important information and reducing the number of computations within the module.

(1) $$X_{reduced}={Conv}_{1x1}\;(X)$$

where $X_{reduced}\;\in \;R^{C^{\prime }\times \;H\;\times \;W}\;$ and $C^{\prime }=C/r\;$ where r is the reduction ratio. This down sample output in $X_{reduced}\;$ is passed through spatial and channel attention modules.

**Figure 3:**
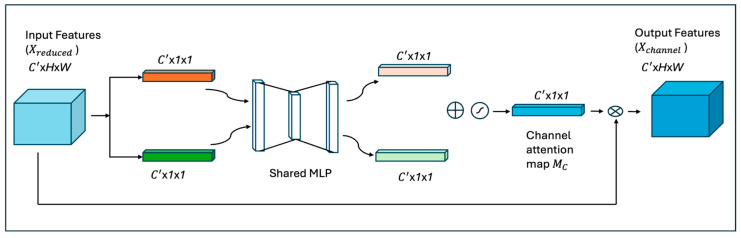
The architecture of the channel attention module.

**Channel attention block:** A channel attention [16] module enhances the prioritized channels, which boosts performance and suppresses channels with redundant information. Channel attention exploits the inter-channel relationship of features. As shown in Figure 3, the first step in channel attention is to apply average pooling and max pooling operations, generating two different spatial context descriptors, as shown in the equations below.

(2) $$F_{avg}^{c^{\prime }}=AvgPool(X_{reduced})$$

(3) $$F_{max}^{c^{\prime }}=MaxPool(X_{reduced})$$

The descriptors are then forwarded to a shared multi-layer perceptron (MLP) with one hidden layer to produce an attention map $M_{c}\in \;R^{c^{\prime }\times \;1\;\times \;1}$. After applying the shared network to each descriptor, we merge the outputs using elementwise summation.

(4) $$M_{c}\left(F\right)=\sigma (MLP(F_{avg}^{c^{\prime }})+MLP(F_{max}^{c^{\prime }}))\;=\sigma ({\;W}_{1}(W_{0}(F_{avg}^{c^{\prime }}))+W_{1}(W_{0}(F_{max}^{c^{\prime }})))$$

where σ denotes the sigmoid function, W0 ∈ Rc′r2× c′ , and W1 ∈ Rc′× c′r2 where $r_{2}$ is the reduction ratio to reduce the parameters in the MLP. MLP weights $W_{0}$ and $W_{1}$ are shared by both descriptors and the ReLU function is followed by $W_{0}$. The final attention map and the input features $X_{reduced}\;$ are multiplied to create the recalibrated features

(5) $$X_{Channel}=X_{reduced}*M_{c}\left(F\right)$$

where $X_{channel}\;\in \;R^{c^{\prime }\times \;H\;\times \;W}$.

**Figure 4:**
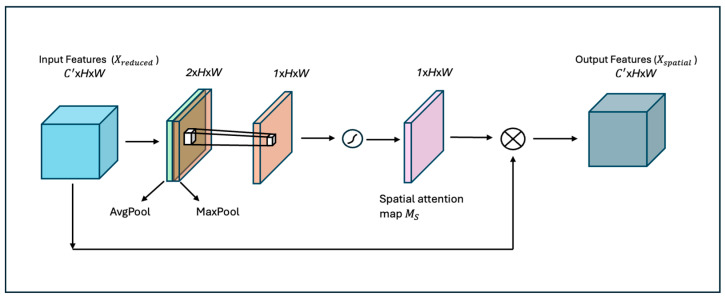
The architecture of the spatial attention module.

**Spatial attention block:** Spatial attention [16] is a mechanism that selectively focuses on specific spatial locations within feature maps, highlighting areas of importance while de-emphasizing others. This approach improves the model’s ability to capture spatial dependencies and allows it to dynamically allocate attention across the feature map’s spatial dimensions (height and width). As shown in Figure 4, the first step in this module is to aggregate the channel information using two pooling operations, generating two 2D maps $F_{avg}^{s}\in \;R^{1\times \;H\;\times \;W}\;$ and $F_{max}^{s}\in \;R^{1\times \;H\;\times \;W}$. $F_{avg}^{s}$ and $F_{max}^{s}$ denote the average-pooled and max-pooled features across the channel. They are then concatenated and convolved by a standard convolution layer, producing an attention map. Overall, the spatial attention map is computed as follows.

(6) $$M_{s}\;\left(F\right)=\sigma \;(f^{7\times 7}({[F_{avg}^{s};F}_{max]}^{s}))$$

where σ denotes the sigmoid function and $f^{7\times 7}$, representing a convolution operation with a filter size of 7 × 7. The attention map $M_{s}$ is used for element-wise multiplication of each channel in $X_{reduced}$

(7) $$X_{spatial}={X_{reduced}\;⊗\;M}_{s}$$

**Output of Hybrid Attention Module:** The final step in this hybrid attention module is to concatenate $X_{spatial},X_{channel}$ along the channel dimension as represented in Equation (8).

(8) $$X_{output}=Concat(X_{channel},X_{spatial})$$

where $X_{output}\;\in \;R^{\;2\;\times \;C^{\prime }\;\times \;H\;\times \;W}$.

Through this module, the attention head module adaptively enhances relevant features, leveraging both channel and spatial contexts while maintaining an efficient parameter count.

#### 2.2.3. Classification Head

After the feature extraction and attention layer, the features are passed through the classification head, as shown in the third block in Figure 2. The classification head contains a Global Average Pooling (GAP) layer, which reduces spatial dimensions of $X_{output}$ by taking the average across each feature map. The GAP operation can be described by

(9) $$F_{GAP}\left[c\right]=\frac{1}{H\times W}\;\sum_{i=1}^{H}\sum_{j=1}^{W}X_{output}[c,i,j]$$

where *H* and *W* are the height and width of feature maps, and c denotes each channel. The pooled feature vector $F_{GAP}$ is then passed through a fully connected layer to classify into normal and glaucoma, as illustrated in Figure 2.

## 3. Results

This section presents the implementation details and results of the proposed framework, along with state-of-the-art models. We compare our proposed model’s performance with normalization-free ResNets and baseline ResNet models.

### 3.1. Training Setting

We trained the models over 100 epochs for optimal performance, incorporating a warm-up period of five epochs and maintaining consistent settings across all experiments. A cosine scheduler was employed alongside the Adam optimizer, using a momentum of 0.9 and a learning rate of 0.00005. We selected cross-entropy loss as the loss function for model training. The fundus images in the experiments were resized to 224 × 224 pixels during model training and we applied data augmentation techniques, such as random cropping, horizontal flipping, and colour jittering at random during training. The models were implemented in PyTorch (2.7.1) .

### 3.2. Test Results

This section presents the results of implementing the new proposed hybrid attention model between feature extractors and classification heads in normalization-free ResNets. We also compared the performance against the baseline ResNet and normalization-free ResNet models. Multiple performance metrics—accuracy (Acc), kappa score, sensitivity (Sen), specificity (Spec), and area under the ROC curve (AUC)—were considered to effectively assess the performance of the proposed model.

Table 1 includes the quantitative performance metrics of models trained and tested on the LAG dataset. The normalization variants of ResNet outperformed the baseline ResNet models. The models trained with the proposed attention module performed better than ResNet and NF_ResNets. NF_ResNets101 with a hybrid attention module with a reduction ratio r = 4 in the attention module achieved the best accuracy of 0.9394 compared to all the other models. The hybrid attention mechanism, along with normalization-free networks, improved the accuracy of the ResNet26 (r = 4) variant from 0.9081 to 0.9364, the ResNet50 variant with r = 4 from 0.9162 to 0.9323, and the ResNet101 variant with r = 4 from 0.9232 to 0.9394 on the LAG dataset. All other performance metrics including sensitivity, specificity, AUC and kappa also improved after adapting the proposed attention module.

Table 2 illustrates our training and evaluation of baseline and proposed models using the EyePACS dataset. Models utilizing the hybrid attention approach outperformed the baseline models across all three variants. Moreover, normalization-free ResNets demonstrated enhanced performance compared to the standard ResNet models. Furthermore, introducing a customized attention module between the feature extractor and the classification head improved performance across all three variants. All the normalization-free variants with a reduction ratio r = 4 achieved the same accuracy of 0.9130 on this dataset. NF_ResNet26 with r = 2 achieved the best sensitivity of 0.9403, Normalization-free variants of resnet50 (r = 2) and resnet101 (r = 2, 4) achieved a similar specificity of 0.9039. Coming to kappa score, all the normalization-free ResNet variants with the hybrid attention module with r = 4 achieved a kappa score 0.8260.

Table 3 shows the performance of the models trained on the BrG dataset. Similarly to results from other datasets, the normalization-free networks combined with the proposed hybrid attention module demonstrate improved performance compared to the baseline ResNet models. Specifically, the NF_ResNet101 model with an attention module with r = 2 achieved an accuracy of 0.8920, outperforming all other variants on the BrG dataset. The NF_ResNet50 with a ratio r = 2 recorded an accuracy of 0.8887, while the NF_ResNet26 with a hybrid module with r = 2 achieved 0.8821. The NF_ResNet101 variant with the hybrid attention module with r = 2 also surpassed other models with a sensitivity of 0.8933, an AUROC score of 0.9494, and a kappa of 0.7842. Additionally, the NF_ResNet50 with the hybrid attention module with r = 2 achieved the best specificity of 0.9139, and NF_ResNet101 with r = 2 achieved the best sensitivity of 0.8933.

Table 4 shows the performance of models trained on the combined datasets using five-fold cross-validation. The NF_ResNet with the proposed hybrid attention modules outperformed the regular state-of-the-art ResNet and NF_ResNet models. Specifically, NF_ResNet50 (r = 2) achieved the highest accuracy of 0.9193, outperforming all other models, including the baseline ResNet50 and NF_ResNet50 models. In addition, NF_ResNet101 (r = 2 and r = 4) achieved the same accuracy of 0.9164, surpassing the performance of the baseline NF_ResNet101 and ResNet101 models.

When comparing the performance reported in Table 4, the NF-ResNet attention models trained on the combined dataset demonstrated slightly lower accuracy than the models trained on each individual dataset, as shown in Table 1, Table 2 and Table 3. However, we expect that the NF-ResNet attention models trained on the combined dataset may provide more reliable and robust performance when applied to images from previously unseen datasets.

Table 5 presents a performance comparison between existing studies and our proposed models. Our models demonstrate competitive performance relative to other approaches, despite being trained from scratch without pretrained weights from ImageNet or other external libraries. Although some previous studies report higher performance on glaucoma datasets, many of these works employ different experimental protocols, including combined multi-dataset training, diverse preprocessing pipelines, and alternative data splits. These variations make direct numerical comparisons challenging.

In contrast, our study adopts a controlled experimental framework to evaluate the effect of the proposed hybrid attention mechanism on NFNet under consistent training conditions. The primary objective of this work is methodological enhancement rather than large-scale dataset optimization. Nevertheless, we have included a literature comparison table to better contextualize our results within the field.

Table 6 compares the performance of the proposed models with existing state-of-the-art (SOTA) models. To ensure a fair comparison, all models were trained from scratch and evaluated using five-fold cross-validation under the same experimental settings. Among the SOTA models, DenseNet121 achieved the highest accuracy of 0.9193. However, all six proposed models outperformed the SOTA models in overall performance.

## 4. Discussion

**Figure 5:**
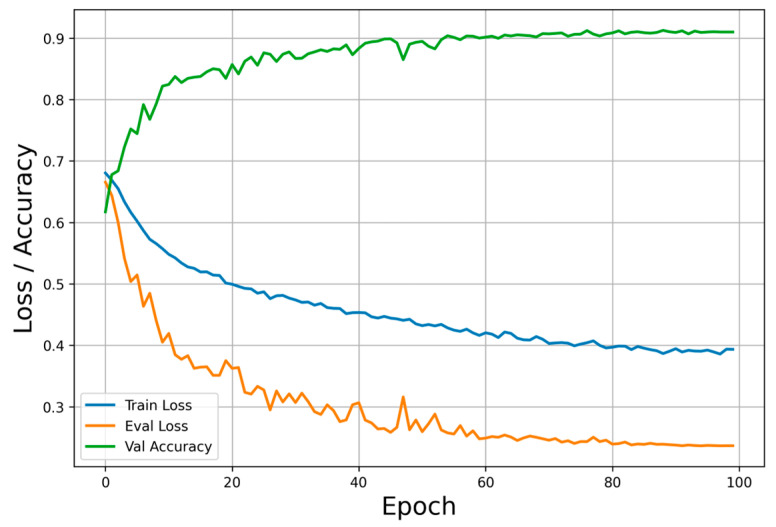
Loss and accuracy curves for NF_ResNet50 (r = 2) during training on the first fold of the combined dataset.

We monitored the variations in training loss, validation loss, and validation accuracy during the training process. Figure 5 illustrates the training curves of these three metrics over epochs for NF_ResNet50 (r = 2), trained on the first fold of the combined dataset under five-fold cross-validation. The training loss, validation loss, and validation accuracy converge with minimal fluctuation after approximately 80 epochs. Similar patterns were observed across the remaining four folds.

**Figure 6:**
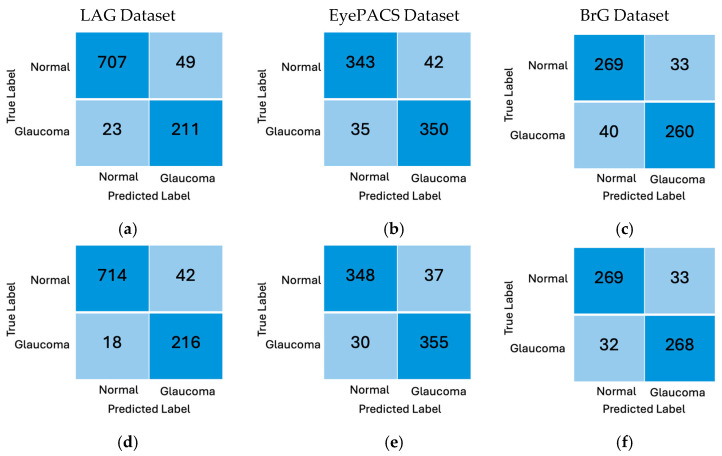
Confusion matrix of best-performing models on each dataset. (**a**) Performance of NF_ResNet101 on LAG dataset. (**b**) Performance of NF_ResNet101 on EyePACS dataset. (**c**) Performance of NF_Resnet101 on BrG dataset. (**d**) Performance of NF_ResNet101 with attention module of r = 2 on LAG dataset. (**e**) Performance of NF_ResNet101 with attention module of r = 4 on EyePACS dataset. (**f**) Performance of NF_Resnet101 with attention module of r = 2 on BrG dataset.

Figure 6 displays the confusion matrix for ResNet101 variants and the best-performing NF_ResNet101 variants with the proposed hybrid attention modules across all datasets, as reported in Table 1, Table 2 and Table 3. The matrices in the first row (a, b, and c) show the performance of NF-ResNet101, while those in the second row (d, e, and f) present the performance of NF-ResNet101 with the proposed hybrid attention modules. The first, second, and third columns display the results for the LAG, EyePACS, and BrG datasets, respectively. Comparing ResNet101 to the NF_ResNet101 with the attention module at r = 2 on the LAG dataset, there are only 42 false negatives, and 18 false positives compared to 49 and 23 on ResNet101.

The other confusion matrices for the EyePACS and BrG datasets clearly indicate a reduction in both false positives and negatives for the NF_ResNet101 with the proposed hybrid attention module, in contrast to the ResNet101 models that do not incorporate the hybrid attention module.

**Figure 7:**
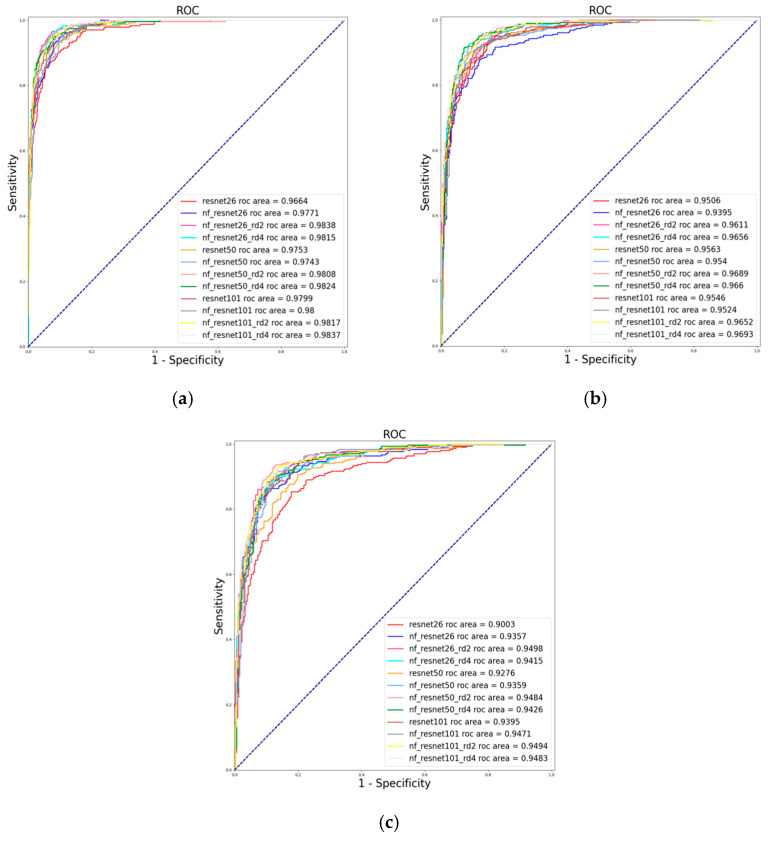
ROC curves for models trained on (**a**) LAG dataset, (**b**) EyePACS dataset, and (**c**) BrG dataset.

Figure 7 shows the ROC curves of the trained models on each dataset for Table 1, Table 2 and Table 3. Figure 7a displays the ROC curves for models trained and evaluated on the LAG dataset. Normalization-free ResNet26 with a hybrid attention module of reduction ratio r = 2 achieved AUC = 0.9838 compared to all the other models trained on the LAG dataset. Figure 7b shows the ROC curves for models trained on the EyePACS dataset. NF_ResNet101 with the attention module using a reduction ratio of r = 2 achieved AUC = 0.9693, which is the best performance on the EyePACS dataset. Figure 7c displays the ROC curves for models trained on the BrG dataset; the NF_ResNet26, along with the attention module having a reduction ratio of r = 2, achieved 0.9498, which outperformed other models on this dataset.

**Figure 8:**
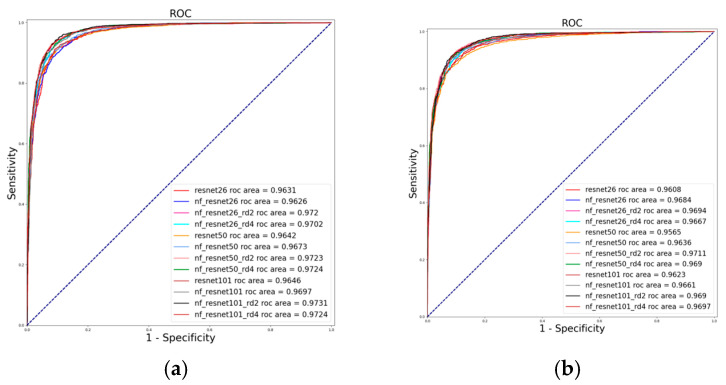

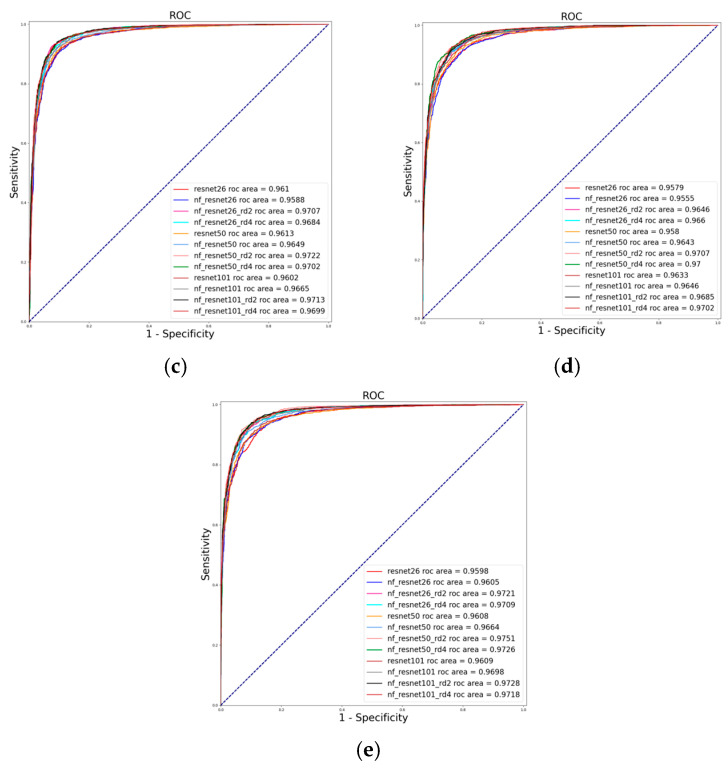
ROC curves for all models trained on the combined dataset: (**a**) Fold 1, (**b**) Fold 2, (**c**) Fold 3, (**d**) Fold 4, and (**e**) Fold 5.

Figure 8 presents the ROC curves of the models trained and evaluated on the five folds of the combined dataset, as summarized in Table 4. Figure 8a shows the ROC curves for models trained and tested on the first fold, where NF_ResNet101 (r = 2) achieved the highest performance with an AUC of 0.9731. For folds 2–5, NF_ResNet50 (r = 2) achieved the highest AUC values of 0.9711, 0.9722, 0.9707, and 0.9751, respectively, demonstrating superior performance across these folds. Overall, the proposed attention-based models consistently achieved higher AUC values than the baseline models across all five folds.

We conducted an error analysis based on the results presented in Table 1, Table 2, Table 3 and Table 4. Among all the proposed attention-based models, comparatively lower performance was observed on the BrG dataset than on the other two datasets. This may be attributed to relatively lower image quality, as the images were captured using an ophthalmoscope attached to a smartphone. In addition, the fundus images in the EyePACS dataset typically cover the entire retinal field, whereas images in the LAG dataset are more focused on the optic disc region. Since the optic disc region contains key clinical indicators of glaucoma, such as the cup-to-disc ratio, the LAG images may provide more detailed structural information relevant to glaucoma classification. Consequently, the proposed attention-based models generated fewer classification errors on the LAG dataset than on the EyePACS dataset.

Classification errors were frequently observed in low-quality images across the three datasets. Furthermore, when the optic disc region appeared excessively bright compared to the surrounding retinal areas, some non-glaucoma images were misclassified as glaucoma. Several non-glaucoma images with peripheral retinal abnormalities outside the optic disc were also incorrectly labelled as glaucoma. We also observed that glaucoma images with low color intensity were sometimes misclassified as normal, whereas non-glaucoma images with uniformly high color intensity across the retina were occasionally predicted as glaucoma.

**Figure 9:**
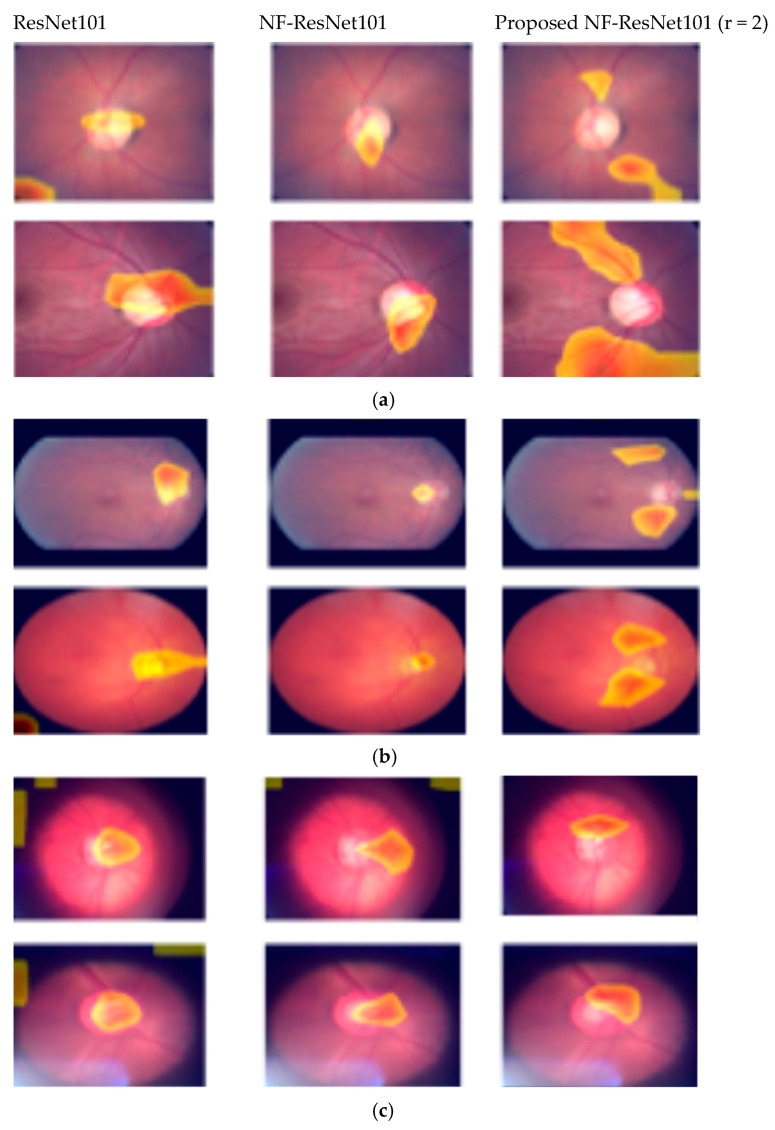
Heatmaps for the ResNet101 variants on various datasets. The heatmaps displayed in the first column belong to the regular ResNet101 models. The heatmaps in the second column belong to their respective normalization-free variants of the respective ResNet101 models. The heatmaps in the third column display the heatmaps from NF_ResNet101 with a hybrid attention module of r = 2. (**a**) LAG Dataset, (**b**) EyePACS Dataset, (**c**) BrG Dataset.

In Figure 9, we present the heatmaps produced by Grad-CAM++ [19] from several randomly selected retina images across all datasets. NF_ResNet101 equipped with the proposed hybrid attention module (r = 2) was utilized to generate the heatmaps. The heatmaps shown in the first, second, third columns correspond to regular ResNet101, NF-ResNet101, and proposed NF-ResNet with the hybrid attention module (r = 2), respectively.

For ResNet101 and NF_ResNet101, the heatmaps were generated from the last convolutional layer, whereas for the proposed NF_ResNet101 (r = 2), the heatmaps were derived from the concatenated outputs of the channel and spatial attention modules.

All normalization-free ResNet variants, together with the proposed hybrid attention module, surpassed all metrics compared to ResNet and normalization-free ResNets. The architectures employing this hybrid attention improve feature maps by utilizing channel and spatial attention mechanisms. This enables the model to concentrate on the most significant channels and spatial areas, thereby enhancing both the relevance of features and the model’s efficiency. Furthermore, attention mechanisms are typically lightweight, introducing only a minimal number of additional parameters relative to the original architecture. By emphasizing key features selectively, they optimize parameter usage and bolster the model’s representational capabilities. This focused approach mitigates the likelihood of overfitting irrelevant details or noise in the training data, thus boosting performance on test data.

Unlike other retinal diseases, such as age-related macular degeneration (AMD), cataracts, and diabetic retinopathy, where key diagnostic features can be identified, glaucoma cannot be diagnosed solely based on fundus images. Clinical diagnosis requires a comprehensive evaluation that includes multiple examinations: tonometry to measure intraocular pressure (IOP), visual field testing to detect peripheral vision loss, optical coherence tomography (OCT) to measure retinal nerve fibre layer (RNFL) thickness, ophthalmoscopy to assess the cup-to-disc ratio (CDR) and neuroretinal rim thickness, pachymetry to measure corneal thickness, and gonioscopy to evaluate the anterior chamber angle. Among these, longitudinal assessment using tonometry, OCT, and visual field testing is particularly important for confirming diagnosis and monitoring disease progression.

Fundus imaging primarily provides structural information regarding the optic nerve head, including CDR and rim thickness. Traditionally, a CDR greater than 0.6 has been used as a threshold to classify glaucomatous eyes. However, reliance on CDR alone has limitations, as some healthy individuals present a relatively large CDR and thin neuroretinal rims without functional impairment. Consequently, fundus image analysis alone is insufficient for glaucoma diagnosis in clinical practice.

Despite these limitations, the proposed deep learning models demonstrated strong performance using fundus images alone, achieving accuracies of 0.8920 on the BrG dataset, 0.9130 on the EyePACS dataset, and 0.9394 on the LAG dataset. Notably, the heatmap visualizations from the ResNet101 model with the proposed hybrid attention module revealed activation patterns concentrated on retinal blood vessels. In contrast, models without the attention mechanism (ResNets and NF_ResNet variants) primarily focused on the central optic disc region as shown in Figure 9. These findings suggest that the hybrid attention module may capture subtle structural cues beyond conventional CDR-based features, potentially including vascular alterations associated with glaucomatous damage.

Nevertheless, given the multifactorial nature of glaucoma, multimodal analysis integrating fundus photography, OCT imaging, and visual field data is likely necessary for robust clinical evaluation. Future work should focus on multimodal fusion approaches to better characterize the relationship between structural changes and functional impairment, and to further investigate the potential association between retinal vascular features and glaucomatous progression.

NF-ResNet26 hybrid attention (r = 4) achieved the best performance on the LAG and EyePACS datasets and the second-best overall performance, with only a marginally higher error rate than NF-ResNet26 hybrid attention (r = 2). However, in clinical setting, NF-ResNet50 hybrid attention (r = 2) would be considered the preferred model, as it achieved the best performance on the combined dataset. This model is expected to provide more reliable and robust performance when applied to input images from previously unseen datasets.

## 5. Conclusions

This study proposes a deep learning model for glaucoma classification from fundus images using a hybrid attention module, which significantly enhances the model’s performance over baseline models when integrated between the feature extractor and the classification head. To thoroughly assess the effectiveness of our proposed module for glaucoma detection in fundus images, we investigated various configurations of Residual Networks (ResNets), including both standard ResNets and normalization-free (NF) ResNets. By evaluating these different variants, we aimed to provide a comprehensive analysis of the performance improvements offered by the proposed hybrid attention module. Our evaluation consisted of an extensive series of experiments conducted on three distinct datasets: LAG, BrG, and EyePACS. The results from these experiments demonstrated that models utilizing normalization-free architectures, when enhanced with our hybrid attention module, consistently outperformed baseline models across all datasets examined. Notably, normalization-free ResNet models exhibited superior performance compared to state-of-the-art ResNet models across all variants tested. For future work, we plan to expand the dataset by adding new retinal images and including data on other retinal diseases.

## Figures and Tables

**Table 1:** Performance of models trained and evaluated on LAG dataset.

Model	Hybrid Attention (r)	Accuracy	Sensitivity	Specificity	F1	AUC	Kappa
	ResNet26 Variants						
ResNet26	No	0.9081	0.8761	0.9180	0.9215	0.9664	0.7572
NF_ResNet26	No	0.9192	0.8889	0.9286	0.9303	0.9771	0.7850
NF_ResNet26	Yes (2)	0.9343	0.9615	0.9259	0.9690	0.9838	0.8299
NF_ResNet26	Yes (4)	0.9364	0.9103	0.9444	0.9445	0.9815	0.8290
	ResNet50 Variants						
ResNet50	No	0.9162	0.8974	0.922	0.9340	0.9753	0.7792
NF_ResNet50	No	0.9232	0.8846	0.9352	0.9289	0.9743	0.7940
NF_ResNet50	Yes (2)	0.9253	0.9615	0.9140	0.9672	0.9808	0.8087
NF_ResNet50	Yes (4)	0.9323	0.9103	0.9392	0.9437	0.9824	0.8192
	ResNet101 variants						
ResNet101	No	0.9232	0.9231	0.9233	0.9483	0.9799	0.7992
NF_ResNet101	No	0.9273	0.9017	0.9352	0.9384	0.9800	0.8060
NF_ResNet101	Yes (2)	0.9374	0.9274	0.9405	0.9532	0.9817	0.8334
NF_ResNet101	Yes (4)	0.9394	0.9231	0.9444	0.9515	0.9837	0.8379

**Table 2:** Performance of models trained and evaluated on the EyePACS dataset.

Model	Hybrid Attention (r)	Accuracy	Sensitivity	Specificity	F1	AUC	Kappa
	ResNet26 Variants						
ResNet26	No	0.8805	0.9013	0.8597	0.8829	0.9506	0.7610
NF_ResNet26	No	0.8766	0.8753	0.8779	0.8764	0.9395	0.7532
NF_ResNet26	Yes (2)	0.8896	0.9160	0.8623	0.8920	0.9611	0.7792
NF_ResNet26	Yes (4)	0.9130	0.9403	0.8857	0.9153	0.9656	0.8260
	ResNet50 Variants						
ResNet50	No	0.8948	0.8883	0.9013	0.8941	0.9563	0.7896
NF_ResNet50	No	0.8974	0.8753	0.9195	0.8951	0.9540	0.7948
NF_ResNet50	Yes (2)	0.9065	0.9091	0.9039	0.9067	0.9689	0.8130
NF_ResNet50	Yes (4)	0.9130	0.9351	0.8909	0.9149	0.9660	0.8260
	ResNet101 variants						
ResNet101	No	0.8792	0.8883	0.8701	0.8803	0.9546	0.7584
NF_ResNet101	No	0.9000	0.9091	0.8909	0.9009	0.9524	0.8000
NF_ResNet101	Yes (2)	0.9039	0.9039	0.9039	0.9039	0.9652	0.8078
NF_ResNet101	Yes (4)	0.9130	0.9221	0.9039	0.9138	0.9693	0.8260

**Table 3:** Performance of models trained and evaluated on BrG dataset.

Model	Hybrid Attention (r)	Accuracy	Sensitivity	Specificity	F1	AUC	Kappa
	ResNet26 variants						
ResNet26	No	0.8355	0.8500	0.8212	0.8384	0.9003	0.6711
NF_ResNet26	No	0.8621	0.8967	0.8278	0.8673	0.9357	0.7243
NF_ResNet26	Yes (2)	0.8821	0.8733	0.8907	0.8813	0.9498	0.7641
NF_ResNet26	Yes (4)	0.8771	0.8600	0.8940	0.8752	0.9415	0.7541
	ResNet50 variants						
ResNet50	No	0.8488	0.8600	0.8377	0.8510	0.9276	0.6977
NF_ResNet50	No	0.8854	0.9033	0.8675	0.8878	0.9359	0.7708
NF_ResNet50	Yes (2)	0.8887	0.8633	0.9139	0.8860	0.9484	0.7774
NF_ResNet50	Yes (4)	0.8870	0.8767	0.8974	0.8862	0.9426	0.7741
	ResNet101 variants						
ResNet101	No	0.8721	0.8567	0.8874	0.8704	0.9395	0.7442
NF_ResNet101	No	0.8787	0.8667	0.8907	0.8775	0.9471	0.7575
NF_ResNet101	Yes (2)	0.8920	0.8933	0.8907	0.8925	0.9494	0.7841
NF_ResNet101	Yes (4)	0.8854	0.8767	0.8940	0.8847	0.9483	0.7707

**Table 4:** Performance of models trained and evaluated on combined LAG, EyePACS, and BrG datasets based on five-fold cross validation.

Model	Hybrid Attention (r)	Accuracy	Sensitivity	Specificity	F1	AUC	Kappa
	ResNet26 variants						
ResNet26	No	0.8976	0.8956	0.8994	0.8887	0.9605	0.7940
NF_ResNet26	No	0.9002	0.8893	0.9094	0.8905	0.9612	0.7989
NF_ResNet26	Yes (2)	0.9154	0.9153	0.9155	0.9080	0.9698	0.8297
NF_ResNet26	Yes (4)	0.9110	0.9100	0.9118	0.9032	0.9684	0.8208
	ResNet50 variants						
ResNet50	No	0.9003	0.8869	0.9116	0.8903	0.9601	0.7990
NF_ResNet50	No	0.9072	0.8996	0.9135	0.8984	0.9653	0.8129
NF_ResNet50	Yes (2)	0.9193	0.9182	0.9202	0.9122	0.9723	0.8375
NF_ResNet50	Yes (4)	0.9155	0.9187	0.9128	0.9085	0.9709	0.8300
	ResNet101 variants						
ResNet101	No	0.9019	0.8880	0.9135	0.8919	0.9623	0.8020
NF_ResNet101	No	0.9104	0.9059	0.9142	0.9022	0.9673	0.8195
NF_ResNet101	Yes (2)	0.9164	0.9158	0.9169	0.9090	0.9709	0.8317
NF_ResNet101	Yes (4)	0.9164	0.9143	0.9181	0.9089	0.9708	0.8316

**Table 5:** Performance comparison with other deep learning methods.

Model	Dataset	Pretrained Weight	Accuracy	Sensitivity	Specificity	F1	AUC
Ensemble using 5 CNNs [13]	BrG	Yes	0.9050	0.8500	0.9600	0.8990	0.9650
ResNet50 [13]	BrG	Yes	0.8810	0.9530	0.8100	0.8890	0.9560
ResNet101 [13]	BrG	Yes	0.8800	0.9100	0.8500	0.8830	0.9490
DenseNet121 [9]	LAG	Yes	0.9381	-	-	0.93049	-
HViTML [17]	LAG	Yes	0.9300	-	-	-	-
DeiT [6]	LAG	Yes	-	-	-	-	0.8800
DG2Net [18]	EyePACS- AIROGS- light-V2	Yes	0.9180			0.9183	0.9190
MaXViT [18]	EyePACS-AIROGS-light-V2	Yes	0.9325			0.9324	0.9325
Proposed Hybrid Attention based on NF_ResNet							
NF_ResNet101 HA (r = 4)	LAG	No	0.9394	0.9231	0.9444	0.9515	0.9837
NF_ResNet50 HA (r = 2)	EyePACS	No	0.9130	0.9351	0.8909	0.9149	0.9660
NF_ResNet101 HA (r = 2)	BrG	No	0.8920	0.8933	0.8907	0.8925	0.9494
NF_ResNet50 HA (r = 2)	LAG, BrG, EyPACS	No	0.9193	0.9182	0.9202	0.9122	0.9723

**Table 6:** Performance of models trained and evaluated on combined LAG, EyePACS, and BrG datasets using five-fold cross validation, trained from scratch without pretrained weights.

Model	Hybrid Attention (r)	Accuracy	Sensitivity	Specificity	F1	AUC	Kappa
ConvNext_Small	-	0.8146	0.8004	0.8264	0.7975	0.8979	0.6265
ConvNext_Base	-	0.8163	0.7987	0.8310	0.7987	0.8989	0.6297
DenseNet121	-	0.9098	0.9107	0.9090	0.9021	0.9680	0.8185
EfficientNetB0	-	0.8079	0.7913	0.8218	0.7899	0.8941	0.6130
EfficientNetB4	-	0.8398	0.8292	0.8486	0.8252	0.9212	0.6773
EfficientNetB7	-	0.8248	0.8155	0.8325	0.8108	0.9007	0.6477
ViT_Small	-	0.7220	0.6869	0.7513	0.6928	0.8002	0.4389
ViT_Base	-	0.7373	0.7127	0.7579	0.7118	0.8131	0.4705
	Proposed Hybrid Attention based on NF_ResNet						
NF_ResNet26	Yes (2)	0.9154	0.9153	0.9155	0.9080	0.9698	0.8297
NF_ResNet26	Yes (4)	0.9110	0.9100	0.9118	0.9032	0.9684	0.8208
NF_ResNet50	Yes (2)	0.9193	0.9182	0.9202	0.9122	0.9723	0.8375
NF_ResNet50	Yes (4)	0.9155	0.9187	0.9128	0.9085	0.9709	0.8300
NF_ResNet101	Yes (2)	0.9164	0.9158	0.9169	0.9090	0.9709	0.8317
NF_ResNet101	Yes (4)	0.9164	0.9143	0.9181	0.9089	0.9708	0.8316

## Data Availability

The original data presented in this study are openly available. The LAG dataset is available at GitHub (https://github.com/smilell/AG-CNN?tab=readme-ov-file, accessed on 8 January 2025). The EyePACS dataset is available via Kaggle (https://www.kaggle.com/datasets/deathtrooper/eyepacs-airogs-light, accessed on 9 January 2025). The BrG dataset is available via Kaggle (https://www.kaggle.com/datasets/clerimar/brasil-glaucoma-brg, accessed on 28 January 2025). The source code is available at: https://github.com/LHNCBC/AI-based-Clinical-Data-Analysis/tree/main/202601_MDPI_Diagnostics_Glaucoma_HA, accessed on 28 January 2025).

## Abbreviations

The following abbreviations are used in this manuscript:

AI	Artificial intelligence
CNN	Convolutional Neural Network
NFNet	Normalizer-Free Neural Network
